# Hidden Information Revealed by Optimal Community Structure from a Protein-Complex Bipartite Network Improves Protein Function Prediction

**DOI:** 10.1371/journal.pone.0060372

**Published:** 2013-04-05

**Authors:** Juyong Lee, Jooyoung Lee

**Affiliations:** School of Computational Sciences, Korea Institute for Advanced Study, Seoul, Korea; University of Zaragoza, Spain

## Abstract

The task of extracting the maximal amount of information from a biological network has drawn much attention from researchers, for example, predicting the function of a protein from a protein-protein interaction (PPI) network. It is well known that biological networks consist of modules/communities, a set of nodes that are more densely inter-connected among themselves than with the rest of the network. However, practical applications of utilizing the community information have been rather limited. For protein function prediction on a network, it has been shown that none of the existing community-based protein function prediction methods outperform a simple neighbor-based method. Recently, we have shown that proper utilization of a highly optimal modularity community structure for protein function prediction can outperform neighbor-assisted methods. In this study, we propose two function prediction approaches on bipartite networks that consider the community structure information as well as the neighbor information from the network: 1) a simple screening method and 2) a random forest based method. We demonstrate that our community-assisted methods outperform neighbor-assisted methods and the random forest method yields the best performance. In addition, we show that using the optimal community structure information is essential for more accurate function prediction for the protein-complex bipartite network of *Saccharomyces cerevisiae*. Community detection can be carried out either using a modified modularity for dealing with the original bipartite network or first projecting the network into a single-mode network (i.e., PPI network) and then applying community detection to the reduced network. We find that the projection leads to the loss of information in a significant way. Since our prediction methods rely only on the network topology, they can be applied to various fields where an efficient network-based analysis is required.

## Introduction

Recent revolutionary advances in protein sequencing technology have made the proteome-scale protein-protein interaction (PPI) data of many species available. For global analysis of large-scale PPI data, the network-based approach has been widely utilized. Ever since the modular nature of biological networks was demonstrated, detecting communities/modules, a set of nodes that are more densely inter-connected among themselves than with the rest of the network, has become one of the most popular approaches for their analysis. Many studies have shown that proteins belonging to the same community within a biological network are more closely related to each other, in terms of functional and physical aspects. Proteins from the same community tend to participate in the same biological process [1], [2], forming protein-complexes more frequently than proteins in different modules.

Utilizing the modular characteristics of the community structure, many researchers have tried to solve a more practical problem, function prediction from biological networks. Annotating protein function is one of the most important issues since the number of species whose whole proteome-scale interaction data is available keeps increasing, and performing large-scale function annotation by experiments is impractical. Conventionally, community-assisted function prediction is carried out by assigning statistically enriched functions, judged by hypergeometric distribution, to all members in the community. However, recent benchmark studies [3], [4] show that community-assisted methods are inferior to simple neighbor-assisted methods, which take into account only direct neighbor information of a target node. Assigning enriched functions to all member proteins of the community generally leads to overprediction, which results in significantly low prediction accuracy. This raises practical concerns regarding the way the community information is used for function prediction on the network.

A number of community-detection methods have been suggested in the past decade [5]–[11]. Among them, the modularity optimization approach is the most widely used to analyze various networks. Modularity is a quality measure to evaluate a given community structure, and it is defined as
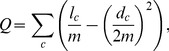
(1)where 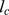 is the number of intra-community edges of community 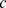, 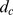 is the sum of degrees of nodes in community 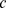, and 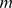 is the total number of edges in the network. Conceptually, the modularity indicates the difference between the fraction of intra-community edges observed and the expected value in a random network. Most community-detection methods have focused on maximizing the modularity of a given network based on the assumption that the community structure with the maximum 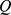 would correspond to the optimal community structure to extract hidden information. However, the meaning of the optimal community structure is dubious.

The relationship between the modularity and the amount of information revealed by the community structure has not been investigated in a systematic way. If there is a positive correlation, it is essential to obtain the community structure with as high modularity as possible to extract maximum information from the network. In practice, however, community structures with reasonably high modularity values are used to analyze biological networks due to the enormous complexity of finding the true maximum modularity solution and limited computational resources. Recently, we have demonstrated that appropriate utilization of an accurate community structure of a protein-protein interaction (PPI) network leads to improved function prediction from the network [12]. This was contrary to the existing benchmark studies [3], [4], where utilization of community information failed to compete with direct-neighbor assisted methods. However, there are a few issues that need to be addressed.

First, many recent studies have highlighted the importance of using bipartite networks, which consist of two types of nodes, for better understanding of complex biological processes. For example, drug-target, disease-gene, and therapy-drug bipartite networks have been widely studied [13]–[17]. Additionally, due to recent advances in experimental methods, protein-complexome network-based studies are also actively investigated [18],[19]. With the bipartite network, there are many unresolved issues. One of them is how to define communities. One way is to deal with the bipartite network directly and allow a community to contain both types of nodes. Another possibility is to project the bipartite network into a single network containing only the type of node of interest. For example, by proper projection, one may opt to construct a weighted PPI network out of a protein-complexome network, and apply the analysis of ref. [12] to extract protein function information. Here, one may want to investigate how much information is gained or lost by projection. Second, the machine-learning based analysis method suggested in ref. [12] is rather complicated. Considering the rapidly increasing size of biological interaction data, a faster and simpler biological network analysis tool with reasonably robust performance is in great demand. Lastly, it is uncertain if improvement to function prediction by incorporating community information depends on the type of a network and/or the function annotation scheme. In ref. [12], only a single PPI network was tested with the function annotation by gene ontology (GO) [20].

**Figure 1:**
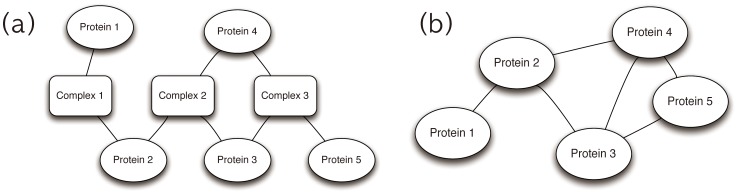
Schematic representation of the protein-complex bipartite network and its protein-mode projection. Schematic representations of (a) the protein-complex bipartite network and (b) its protein-mode projection are shown. In the protein-mode projection, two proteins are connected if they participate in forming at least one identical complex.

In this study, we address the above issues by performing community-assisted function predictions of the protein-complex network of *Saccharomyces cerevisiae* [18], a bipartite network consisting of proteins and complexes (see Fig. 1 for schematic representation of the network), with MIPS function annotations [21]. The predictions were carried out by using a machine-learning based method as well as a newly devised simple screening method. The community structure of the network was identified by the Mod-CSA method, a modularity maximization algorithm based on the conformational space annealing (CSA) global optimization algorithm. We observe that Mod-CSA finds higher modularity solutions than the simulated annealing (SA) method, which has been considered the most accurate until recently requiring less computational time regardless of the network size [22]. In the benchmark test with small networks, a number of independent CSA runs all converged to identical solutions, which coincide with the known optimal solutions found by the exact method [22], [23].

We observe that the highest modularity yielding community structure obtained by CSA is functionally more coherent than sub-optimal ones found by SA, when judged by the number of enriched functions. We compared our community-assisted function prediction methods with two widely used neighbor-assisted methods, 1) neighbor counting (NC) [24] and 2) Markov random field (MRF) [25]–[27]. The result shows that the efficiency of the function prediction can be improved significantly and maximally by using the community-assisted methods with the highest modularity community structure. We find that projecting the original bipartite network of a protein-complexome network into a PPI network leads to the loss of information in a significant way. There are multiple ways to use the community information of a bipartite network and our claim that a community structure can facilitate discovery of additional information from a network appears to be universal across variations in the type of networks and the function annotation scheme.

## Results and Discussion

### Community Detection of the Yeast Protein-complex Network

**Figure 2:**
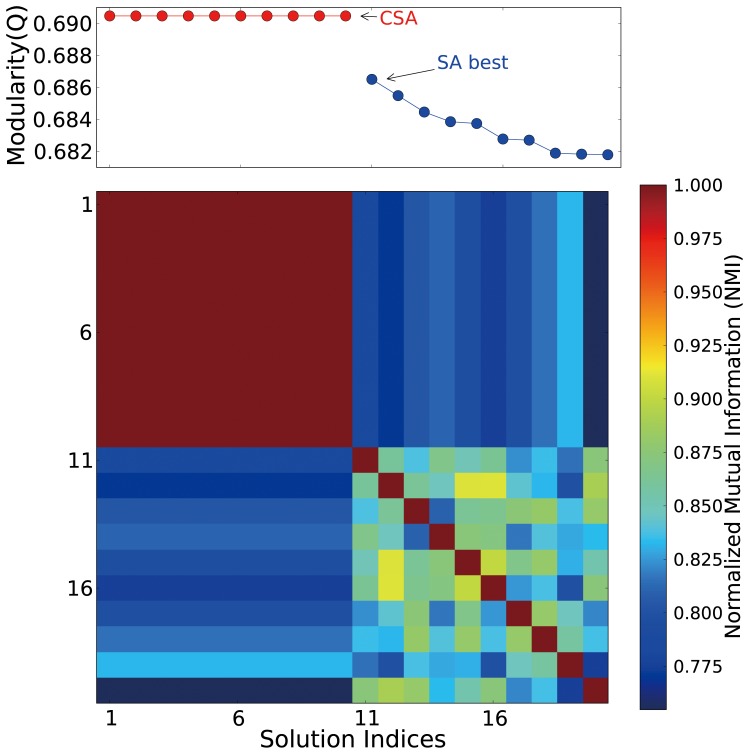
Modularities from the 20 best CSA and SA simulations and the pairwise normalized mutual information. Top panel shows the 20 best modularity values obtained by CSA (10 red circles) and SA (10 blue squares). Note that the 10 best CSA solutions are identical to each other with Q = 0.6905, while none of the 10 best SA solutions are identical to each other; all have lower modularity values than 0.6905.

For the community detection of the yeast protein-complex network with modularity optimization, we used the conformational space annealing (CSA) and simulated annealing (SA) methods [28]. Due to the stochastic characteristics of both methods, we performed 10 independent runs for each method. The best modularity value is recorded for each run, and the 20 best ones are shown in Fig. 2. All CSA simulations provide the identical optimal community structure with the modularity of 0.6905. Based on our previous benchmark result [22], this convergence strongly suggests that this community structure corresponds to the optimal and true maximum modularity solution. On the other hand, the 10 best SA simulations result in various community structures, all of which have modularity values less than 0.6905. The maximum and average modularity values from the 10 best SA runs are 0.6865 and 0.6835, respectively. The apparent gap between the best modularity values indicates that there exists significant difference in search efficiency between CSA and SA. Statistical properties of the modularity optimization results are listed in Table 1. In terms of the number and the sizes of communities, the differences are not significant. The optimal CSA solution divides the network into 24 communities and the average number of communities found by SA is 27.1 with a standard deviation of 3.8.

**Table 1:** The summary of 10 community detection simulations for SA and CSA.

	CSA	SA
*Q_max_*	0.6905 (0.000)	0.6835(0.002)
Number of communities	24 (0)	27.2 (3.83)
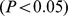 Number of enriched functional clusters	480 (0.0)	427.3 (21.4)

For each simulation, the best Q value (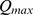) obtained is recorded. Standard deviations are shown in parentheses. It should be noted that all 10 CSA runs provide the identical best solution with 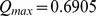.

Qualities of community structures are investigated using a hypergeometric P-value analysis to detect meaningful functional clusters. For each community, all MIPS functional annotations [21] of protein nodes are identified and the P-value of each annotation is calculated from all constituent proteins. The annotation’s hypergeometric P-value, probability of observing at least 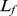 proteins with the annotation 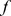 from a community of size 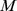, is calculated as
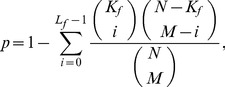
(2)where 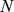 is the number of proteins in the network and 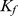 is the number of proteins with 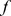 in the network. If the P-value of an annotation is less than a threshold value, the group of proteins associated with that annotation in the same community is considered as a meaningful functional cluster irrespective of connectivity. Since a protein may have multiple functional annotations, the meaningful functional clusters of a community may overlap, and some proteins may not belong to any functional cluster. This should be contrasted to the fact that communities defined in this study form a non-overlapping partition of the projected protein-protein network. It should be noted that functional clusters do not span multiple communities.

**Figure 3:**
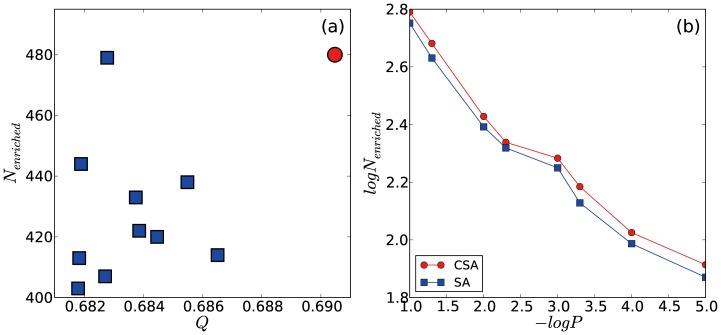
Modularity versus the number of enriched functional clusters, and its dependence on P-value. (a) Best modularity values obtained by 10 CSA simulations (red circle) and 10 SA simulations (blue squares) against the number of enriched functional clusters with the P-value threshold of 0.05 are shown. It should be noted that 10 best solutions by CSA provide an identical solution implying that it may correspond to the true optimal solution. (b) Log-log plot of P-value threshold and the number of enriched functional clusters are shown. The highest modularity community structure obtained by CSA yields larger numbers of enriched functional clusters than those by SA regardless of the P-value threshold used.

The average numbers of meaningful functional clusters obtained by CSA and SA with P-value threshold of 0.05 are 480 and 427.3 (with a standard deviation of 0 and 21.4), respectively, as shown in Fig. 3-(a). The number of meaningful functional clusters from SA ranges between 403 and 479, which may cause problems in the reproducibility of the method. However, CSA provides a converged optimal community structure, which reveals many more meaningful functional clusters. We checked that this result is insensitive to the selection of the P-value by varying the P-value threshold between 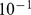 and 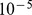. The average number of meaningful functional clusters from SA is consistently lower than that from CSA, as shown in Fig. 3-(b). In conclusion, CSA finds the optimal community structure of the yeast protein-complex network in terms of the modularity, and this community structure reveals more functional information of proteins than the sub-optimal ones generated by SA.

**Figure 4:**
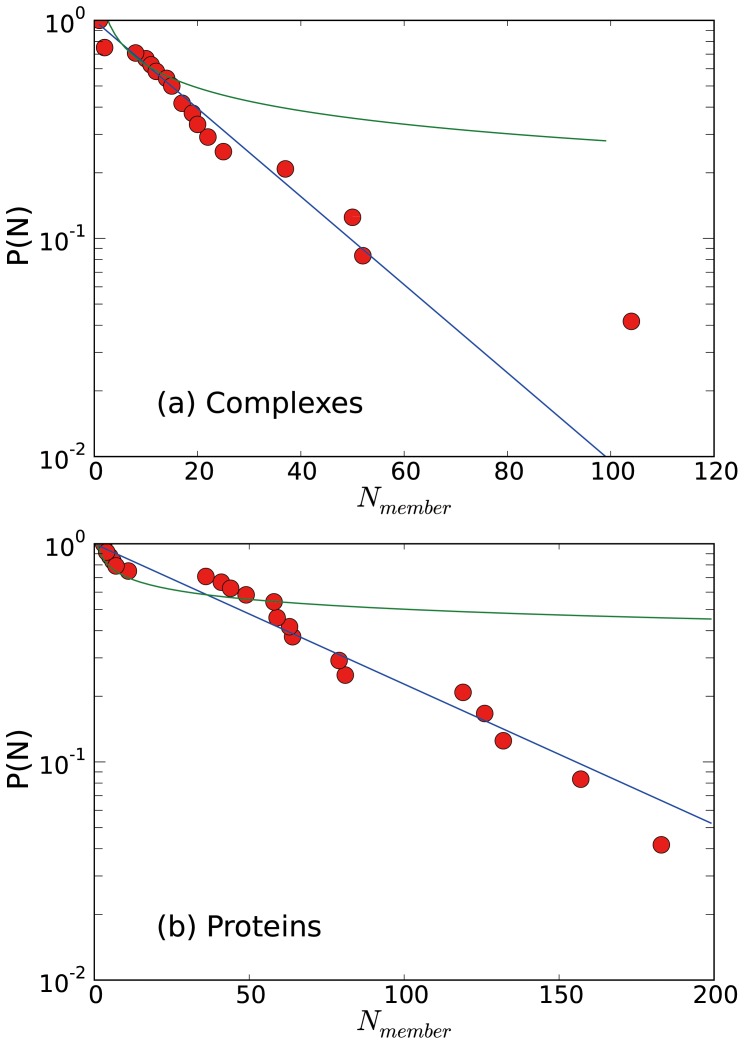
Distributions of the number of complexes and proteins in a community. Distributions in number of (a) complexes and (b) proteins are shown. Here, 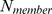 corresponds to the number of complex/protein nodes belonging to a community. Symbols correspond to the cumulative distribution of complex/protein nodes. Lines correspond to the exponential and the power-law fittings, and the data fits better by exponential lines.

Using the optimal community structure, we investigated the distribution in numbers of complexes and proteins belonging to a community, as shown in Fig. 4. It is clear that the distributions are more consistent with the exponential distribution than with the power-law distribution, as is frequently reported in diverse biological networks [29], [30]. It should be noted that the cumulative distribution of an exponential/power-law distribution also follows the exponential/power-law distribution in the continuum limit. Only the number of complexes in the largest community seems to deviate from the exponential distribution significantly. Previously, it was observed that the degree distribution of nodes also follows the exponential distribution [18]. This indicates that the exponential distribution is also preserved in the coarser organization of the network. As discussed in the previous study [18], the exponential distribution is due to the nature of the experimental method used, TAP-MS, which detects actual physical complexes of proteins rather than collecting their binary interactions. Generally, the power-law distribution implies the existence of extreme cases, such as hub proteins with extremely large numbers of partners. If a protein-protein interaction network is constructed from binary interaction data, there is no theoretical limitation in the number of interacting partners, which allows for the formation of a hub node or a hub community. However, in the actual cellular environment, the size of a community, the number of densely inter-connected proteins or complexes, is limited by several physical factors, which prevent the formation of extreme cases. Limiting physical factors include the rate of protein transport, the size of a cell, and the crowding effect of the cellular environment.

### Network of Communities

**Figure 5:**
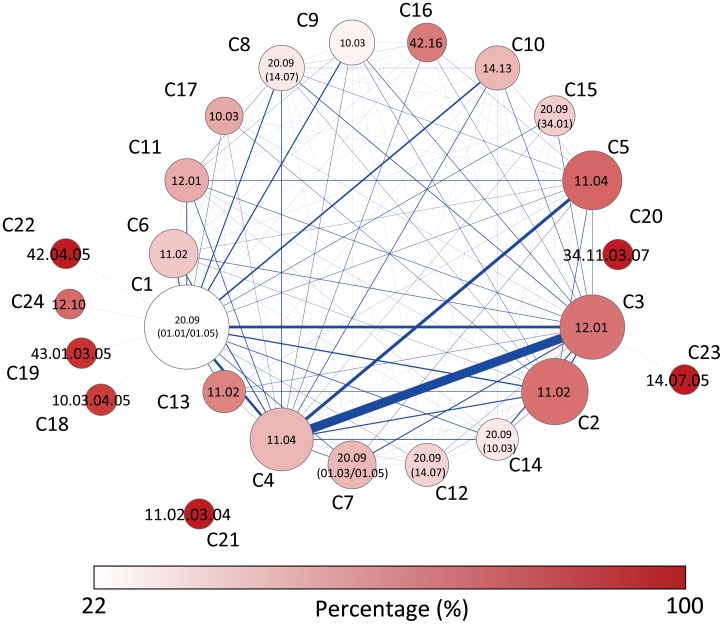
Network between communities of the yeast protein-complex network. The size of each community node represents the number of proteins in the community, and the opacity of the node represents the percentage of proteins with the most observed function in the community, labeled at the center. The width of the edge corresponds to the number of inter-community links.

To obtain a general picture of the yeast protein-complex network, we constructed a reduced representation of the network, the network of communities, as shown in Fig. 5. The full list of the functional compositions of identified communities is provided as supporting information (Table S1 and Table S2). The size of a node represents the number of proteins in the community, and its opacity represents the fraction of proteins sharing the most observed function in the community, labeled at the center. The width of the edge represents the total number of inter-community links. The most strongly interacting communities, C3, C4, and C5, are mainly involved with the translation processes, *12.01 ribosome biogenesis* and *11.04 RNA processing*. We observe that most functionally specialized communities are relatively small, and they are connected with only one or a few other communities. For example, C18, C19 and C22 are connected only to C1, and over 90% of member proteins in these communities are involved in the process shown. These three communities are involved with cell division processes: *42.04.05 microtubule cytoskeleton*, *43.01.03.05 budding, cell polarity and filament formation* and *10.03.04.05 chromosome segregation/division*. We find that these communities are distinctly separated from the rest of the network since cell division is rather specific compared to other metabolic processes in spatial and temporal aspects. The other small and specific communities, C20, C21, and C23, appear to play the role of the regulatory switch of the transcription process. C20 is connected only to RNA processing communities C4 and C5, and it is occupied by *34.11.03.07 sex-specific proteins*. C21 interacts only with C4, and it consists of proteins performing transcriptional control, *11.02.03.04*. C23 is related with the ubiquitination process, *14.07.05*, tagging unneeded proteins to be recycled, which is an error correction step.

In 6 out of 24 communities, transport proteins are closely coupled with proteins that require transport for proper functioning. The *20.09 transport routes* annotation dominates 6 communities: C1, C7, C8, C12, C14, and C15, but its composition fractions are relatively low. The lowest and the highest fractions are 22% and 43%, observed in C1 and C7 respectively. Note that the largest community C1 is *the least concentrated* in terms of its top function, represented by the pale color of the node in Fig. 5. The second most observed function (shown in parentheses), after *20.09* differentiates each community. C1 and C7 are mainly related with metabolism processes, *01.05 C-compound and carbohydrate metabolism*. Additionally, *01.01 amino acid metabolism* and *01.03 nucleotide/nucleoside/nucleobase metabolism* annotations are observed in C1 and C7, respectively. Many proteins from C8 and C12 are involved with the post-translational modification, *14.07 protein modification*, which requires the transport of substrates such as phosphates for phosphorylation and carbohydrates for glycosylation.

### Protein Function Prediction on the Protein-complex Network

Here, we propose two ways to incorporate community structure information into protein function prediction, 1) simple screening over neighbor-assisted predictions and 2) construction of a machine learning algorithm by the random forest (RF) method [31], which utilizes the community structure of a network as input features. The first scheme is based on the assumption that the more a function of a target node’s neighbors is enriched in its community, the more likely it is that the node possesses the function. In this simple screening, the function prediction of a node is performed by taking the intersection of a neighbor-assisted prediction and screening by functional enrichment in terms of P-value.

The second scheme employs RF, which takes advantage of examples to capture hidden and nonlinear relationships between the presence of a function on a node and its local/global properties generated from the network topology. We compared our community-assisted function prediction methods with two widely used neighbor-assisted methods, the neighbor counting (NC) method [24] and the Markov random field (MRF) method [25]–[27]. To evaluate the performances of prediction methods, leave-one-out cross validations were carried out. For each method, the functions of each protein node are assumed to be unknown and they are predicted out of all annotated functions from its direct neighbors, iteratively.

#### Simple screening

**Figure 6:**
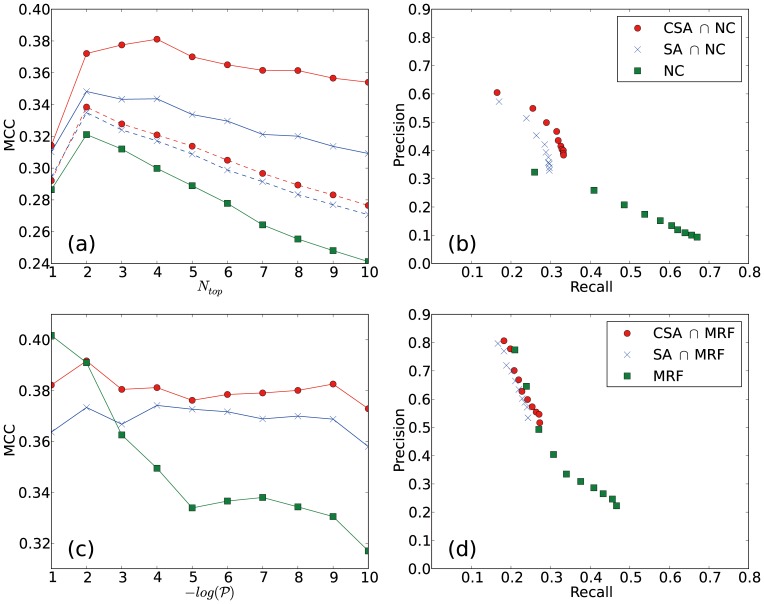
Prediction results of two neighbor-assisted methods and their screening counterparts. Neighbor counting (NC) results (green squares) are shown in (a) and (b), and Markov random field (MRF) results (green squares) are shown in (c) and (d). Corresponding simple screening results using the P-value threshold of 0.05 are shown in red circles (CSA) and blue crosses (SA). The variation of Matthews correlation coefficient (MCC) along with the number of predictions made by the neighbor assisted methods, (a) and (c), and the precision-recall plot, (b) and (d), are shown. Dashed lines in (a) represent the results obtained by using the community information detected in the weighted projection of the bipartite-network. Note that the simple screening result using the optimal community structure obtained by CSA improves the prediction quality for all cases tested (except 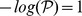 for MRF) in terms of the MCC measure.

Performances of prediction methods are shown in Fig. 6 by using the Matthews correlation coefficient (MCC), (a) and (c), and the precision-recall (PR) curve, (b) and (d). The P-value threshold for screening was set to 0.05. MCC values are measured along the thresholds of the neighbor-assisted methods, i.e., the rank (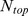) of observed functions in NC and the probability (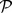) threshold for MRF. In the MCC plot, Fig. 6-(a) and (c), the increase of 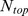 and 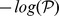 values corresponds to the increase of positive predictions, which leads to higher coverage and lower accuracy predictions.

By screening with the meaningful functional clusters identified from the CSA community structure, the MCC values of NC and MRF improve throughout the variation of the threshold value, except for the case of MRF with 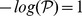. The average MCC values of NC and MRF are 0.279 and 0.344, while the corresponding values obtained by the combination of communities from SA and CSA are 0.329, 0.367, and 0.369, 0.380, respectively. From the PR curves, Fig. 6-(b) and (d), it is clear that the screening helps to identify more accurate predictions, which shifts the neighbor-assisted results (green squares) to higher accuracy regions (red circles and blue crosses). In other words, the use of meaningful functional clusters effectively reduces false positives. For example, when we combine the best NC result of 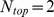 with screening using the optimal CSA community structure, the precision improves by 29.0%, and the recall drops by only 15.4%. As the threshold value increases, MCC values of the neighbor-assisted methods decrease sharply, implying that they are efficient only for predicting a small number of evident functions and not suitable for an extensive prediction. However, together with the community structure information, the quality of function prediction in terms of the MCC value appears to be relatively well preserved or deteriorates in a much slower manner. For the original NC method, the MCC value linearly drops from 0.321 to 0.241 as 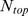 increases from 2 to 10. When combined with suboptimal community structure information from SA, the overall quality still improves, and the gap between the best MCC of 0.348 and the worst MCC of 0.309 is reduced by half. With the optimal community information from CSA, the MCC value increases until 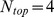 and decreases slowly thereafter.

MRF clearly outperforms NC as reported in previous studies [32]–[34]. Starting from 0.401, the MCC value of the native MRF method decreases dramatically to 0.335, and it forms a plateau after 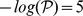. However, when combined with the community information, the quality of the prediction improves and stays invariant under the variation of the parameter. In practice, this invariance is quite advantageous since it allows one to choose between precision and recall according to the situation without sacrificing the prediction efficiency.

We have examined the case of 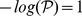, where the MCC of MRF is higher than that of MRF with screening. We find that this result is mainly due to rather uniformly spread and highly observed function annotations. More than 78% of the true positives by MRF come from top 10 most observed functional annotations. Since many of these functional annotations are not significantly clustered at the community level identified in this study, they are screened out when MRF predictions are combined with the community structure information. This indicates that the resolution of the obtained community structure may not be suitable for describing the grouping of these annotations, and there must be a relationship between the resolution in the community detection and the resolution in the distribution of functions on the network. Therefore, it is essential to find the proper resolution of a community detection method to extract maximum information about a particular function of interest; our focus in this work was that we can obtain more information about hidden attributes of nodes, function annotations in this work, when a community structure is considered, which can be determined with the proper resolution for the attributes of interest.

#### Random forest

**Figure 7:**
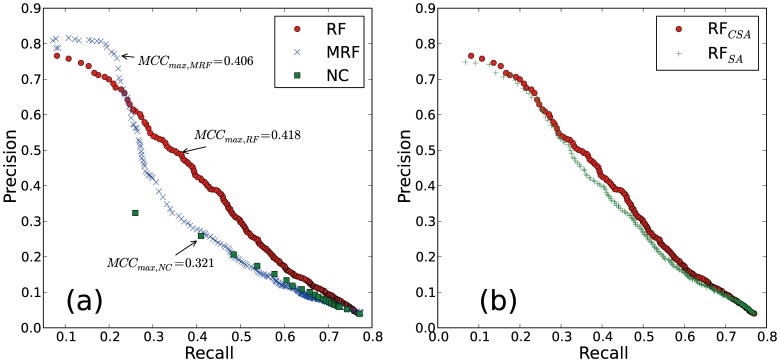
Precision-recall curves for various methods. Precision-recall curves for neighbor counting (NC), Markov random field (MRF) and the current study (RF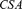) are shown in panel (a). MCC maximum values are indicated by arrows. MRF provides quite accurate prediction for the low recall region (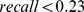), which corresponds to true positive predictions for the top 10 highest background functions. In panel (b) RF using community structure from SA (RF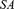) is shown along with the CSA data.

The AUC values and the maximum MCC values obtained from RF and the neighbor-assisted methods are listed in Table 2. In terms of the AUC value, RF outperforms the neighbor-assisted methods. The best result is obtained when the optimal community structure from CSA is used to generate input features for RF. The AUC value of RF using the CSA result is higher than that of MRF and NC by 11.1% and 62.1% respectively. RF using CSA result shows the highest maximum MCC value of 0.418 with (recall, precision) = (0.362, 0.493). PR curves of the prediction results from neighbor-assisted methods and RF are shown in Fig. 7. Note that the maximum recall is observed at 0.774 not 1.0, which reflects the cases where none of a protein node’s direct neighbors possess the functions of the node. Overall, RF outperforms the neighbor-assisted methods tested except in the high-precision, low-recall region of MRF. As discussed above, MRF performs well for high-background and widespread annotations that may not be properly featured by the community structure, presumably due to the resolution limit of the modularity. Therefore, the usage of the community structure information may deteriorate the prediction quality for these annotations. On the other hand, RF performs well for the prediction of annotations, which are less populated but specifically clustered in the network.

**Table 2:** Prediction efficiencies of random forest and two neighbor-assisted methods.

Measure	RF with CSA	RF with SA	MRF	NC
AUC	0.300	0.293	0.270	0.185
*MCC_max_*	0.418	0.401	0.406	0.321

Prediction efficiencies are measured by AUC (area under the PR curve) and the maximum value of MCC (Matthews correlation coefficient) defined in Eq. 9. Methods tested are NC (Neighbor counting), MRF (Markov random field), and two random forest methods combined with community structures by CSA and SA.

### The Effect of Projection to a Protein-protein Interaction Network

A weighted projection of a bipartite network is often considered a fair substitute for the original bipartite network, presumably with little information loss. To investigate the effect of the projection on the performance of the function prediction methods, we performed a simple screening analysis using the communities identified from a weighted PPI network generated by the projection of the full protein-complex network. The weight of an edge between two proteins in the reduced network is assigned by the number of common complexes shared by the two proteins. The MCC plot of the simple screening using the NC method and the communities from the projected network is shown as dashed lines in Fig. 6-(a). The plot clearly shows that the degree of prediction improvement with the projected network is significantly less than with the bipartite network. The maximum and average MCC values with the CSA result decreased from 0.380 and 0.367 to 0.338 and 0.304.

The less optimal performance with the projected network may be attributed to the much larger number of assigned edges between protein nodes. The average degree of protein nodes in the bipartite network and the conventional protein-protein interaction (PPI) network generated by the collection of binary interactions, from [12] are 4.51 and 8.92, respectively. However, the corresponding value of the projected network is 63.5. This large difference in the average degree of protein nodes implies that the projection of the protein-complex bipartite network can introduce many false-positive interactions and the information of the original bipartite network is somewhat diluted by the projection. This suggests that communities detected from the bipartite network and from its projected network can be significantly different from each other, and care should be taken to choose an appropriate type of a network to extract maximum information.

### Improvement in Protein Function Prediction by Community Information

Improvement in the protein function prediction with both methods shows that there are multiple ways to utilize community structure. The difference in performance between methods shows that there is a trade-off between performance and computational cost. Our study can be a guideline to select a method, which is fit for a desired purpose. The advantage of the new simple screening method over the RF method is that it is faster and can readily be performed with publicly available resources without additional data handling. For a given community structure, the functional enrichment of each community can be obtained from various existing web-servers [35]–[37]. Taken together with our previous study [12], the results of this study indicate that information mined from a network is universally enhanced when community structures are used, regardless of variations in the type of network and the function annotation scheme.

It should be noted that the improvement of prediction quality in both community-assisted schemes is more prominent when the optimal community structure generated by CSA is used, rather than the sub-optimal one from SA. Based on the PR data, the optimal community structure information improves both the precision and the recall of the function prediction. In conjunction with the number of meaningful functional clusters, we have shown (above) that the optimal community structure with the maximum modularity reveals more functional information than sub-optimal ones. Many previous studies, which utilize the community structure of a biological network paid little attention to the quality of the community structure. However, as shown in this study, the amount of information extracted substantially depends on the quality of the partitioning. Therefore, when performing the community analysis of a biological network, it is essential to work with the optimal community structure with maximum modularity. For this purpose, CSA is the method of choice. In a previous study, we have shown that CSA can obtain optimal community structures of networks with up to 2,000 nodes within reasonable computational time and much faster than SA [12], [22]. For the community detection of the yeast protein-complex network investigated in this study, it took 3 hours with 8 processors on average for a CSA simulation while it took 3 days with a single processor for a SA simulation. The parallel implementation of CSA allows one to deal with much larger networks, which are inaccessible to SA.

It should be emphasized that our method improves the function prediction efficiency *without additional* source of information. Many existing function prediction studies have relied on utilizing additional external information to improve the prediction, such as the reliability of the link presumed by the experimental method [32], [33] or the ortholog information across the networks of several species [38], [39]. The current method can be combined with other existing methods in a straightforward manner, which can lead to additional improvement.

## Materials and Methods

### Bipartite Protein-complex Network and Function Annotations

We have constructed a bipartite network consisting of complexes and their component proteins generated from genome-wide characterization of protein complexes of the yeast *Saccharomyces cerevisiae* [18], [40]. The original set consists of 491 protein complexes and 1,491 component proteins. When island nodes not connected to the main network are removed, the network contains 476 protein complexes and 1,452 component proteins. Community structure was determined by modularity optimization approach with the modified modularity measure (see below) for the bipartite network [41].

To perform function prediction on the network, we generated a protein-mode projection, where two proteins are connected if both of them participate in forming at least one identical complex. Schematic representations of (a) the bipartite network and (b) its protein-mode projection are shown in Fig. 1.

Throughout the function prediction calculations carried out in this study, we used the MIPS FunCat annotations [21]. MIPS FunCat annotations are organized in a hierarchical manner and each level is separated by a dot, e.g. *01 metabolism* is a representative at the most coarse level, and *01.01.03.02.01 biosynthesis of glutamate* belongs to the most specific level of FunCat. Among 1,360 MIPS function annotations, we focused on the specific functional terms finer than the third level, with at least three dots in the annotation, which results in 228 annotations. We note that proteins without any known functional annotations were excluded from the function prediction experiment.

### Community Detection via Modularity Optimization

A modified modularity measure is used [41] to detect the community structure of the bipartite network. Due to the explicit dependence of the modularity on the null model, a randomly re-wired network, Eq. 1 of the original modularity is modified as
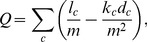
(3)where 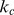 and 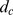 are the sum of degrees of two types of nodes (i.e., proteins and complexes) in community 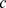.

Here, we briefly review two modularity optimization algorithms, simulated annealing (SA) and conformational space annealing (CSA). SA is a stochastic optimization method. It is rather slow but has been considered as the most accurate method until now. Here, we followed the implementation of previous studies [28], [42]. Initially, the simulation starts at a high temperature, 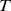, to sample wide regions of the solution space and to avoid getting trapped in local maxima. As the simulation proceeds, 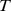 is slowly decreased to focus on sampling high modularity regions. At a given 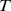, a set of moves including 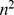 single-node moves and 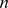 collective moves consisting of random merges and splits of communities, is carried out, where 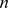 is the total number of nodes in the network. For each trial move, if Q increases (from 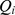 to 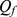), the move is always accepted. Otherwise, it is accepted with the probability of 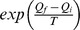. After a set of moves is tried, 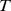 is reduced to 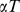, where 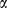 = 0.995.

CSA is a global optimization algorithm based on the genetic algorithm (GA). CSA uses a population of candidate solutions, which evolves toward better solutions through cross-over and mutation followed by local optimization. In CSA, each solution is assumed to represent a hyper-sphere in the solution space with the radius of 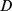. 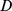 determines the diversity of sampled solutions, and it starts as a large value, which decreases as the search proceeds. 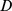 is equivalent to 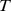 in SA. When 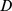 is large, candidate structures must be far apart in solution space; as 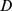 shrinks, good candidate solutions are further fine-tuned. The distance between two community structures is measured in terms of normalized mutual information (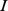) [43]. For two given partitions of 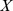 and 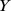, 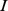 is defined as
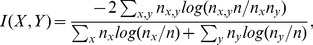
(4)where 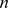 is the total number of nodes, 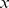/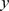 refers to a community from 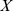/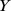, 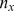/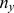 is the number of nodes in 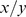, and 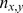 is the number of nodes shared between 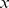 and 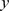. If 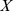 is identical to 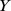, it is easy to show that 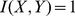.

For cross-over, we introduced two operators, a simple copy and a divisive copy. In both operators, one community is randomly selected from the source solution and it is transferred into the target solution by changing community identifiers of nodes in the target according to the selected community structure of the source. The rationale of these operators is that the community index of an individual vertex is only a dummy variable whereas a well-defined community should be conserved and transferred into new solutions. In the simple copy, the community identifier of the selected community of the source is transferred to the target unchanged. On the other hand, in the divisive copy, a new community identifier not present in the target is given to the selected community. Using only one kind of the two operators results in getting trapped in local maxima and lower modularity solutions.

For local optimization of a trial solution, a quench simulation, which accepts only modularity improving moves, equivalent to SA at 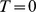, was performed consisting of 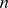 individual node moves and 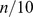 collective moves, random merge and split. For CSA simulations, the first cycle of CSA starting with 50 random solutions is carried out until the population reaches a deadlock in terms of a relevant signal. When this happens, the second cycle is performed with the population increased to 100 to sample other regions of the solution space not properly searched in the first cycle. Generally, the size of the final population should be set to a rather large number as the complexity of the problem grows. In this study, it is set to 100. Recently, we compared the performance of CSA to that of SA [22]. The result shows that CSA consistently finds higher modularity community structures using fewer computational resources than SA. More detailed descriptions and benchmark results of the algorithm can be found elsewhere [22], [44]–[46].

Due to the stochastic nature of the two methods, we have performed 10 independent modularity optimization runs for each method. As discussed in the Result section, all 10 CSA runs provide an identical community structure as the best solution whereas the 10 SA runs yield various suboptimal solutions. Among the 10 best solutions from SA, the highest modularity community structure is used for protein function prediction.

### Protein Function Prediction

#### Protein function prediction via neighbor-assisted methods

In this study, two widely used neighbor-assisted protein function prediction methods are utilized, a) *Neighbor counting* (NC) [24] and b) *Markov random field* (MRF) [25]–[27]. In NC, 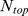 most observed functions among direct neighbors of the target protein are predicted as its functions. In the original implementation, 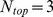 was used. Here we have evaluated the performance by varying 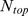 from 1 to 10. MRF is a probabilistic method, and it relies on the Markovian assumption that the function of a protein depends only on the states of its direct neighbors. In MRF, the probability 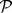 that a protein 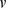 is associated with a function of its neighbors is defined as
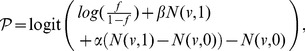
(5)where *logit(x)* is the logistic function of 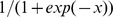. 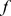 is the background fraction of the function under consideration. 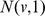 and 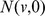 denote the number of direct neighbors with and without the function, respectively. 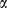 and 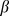 are free parameters, and in this study, we followed the implementation of Karaoz et al. [26] where 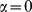 and 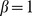 is used. By varying the probability 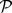, the balance between precision and recall can be adjusted with lower 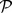 values resulting in higher precision and lower recall prediction.

#### Protein function prediction by screening neighbor-assisted predictions

In this study, we propose a simple screening scheme over neighbor-assisted predictions. For each prediction by a neighbor-assisted method, the function’s hypergeometric P-value, the probability of observing at least 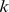 proteins with the function from the community of size 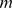, is measured as
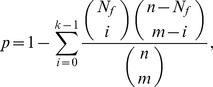
(6)where 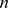 is the number of proteins in the network, 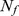 is the number of proteins with the function in the network, and 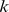 is the number of protein with the function in the community. Low P-values indicate that it is unlikely that 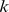 separate proteins all associated with an identical function belong to the current community by chance. P-value can be used as an indicator to identify, which results from neighbor-assisted predictions are more reliable. Using a P-value threshold of 0.05, neighbor-assisted predictions with 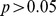 are screened out. Note that the result is rather insensitive to the variation of the P-value threshold.

#### Protein function prediction by random forest

To capture nonlinear relationships between the presence of a particular function annotation on a protein node and its local/global properties from the network topology, we employed the random forest (RF) machine learning algorithm and performed the leave-one-out cross validation with MIPS function annotations [12]. We deleted each target node individually from the network and attempt to predict its functions using the network information of the remaining 1,490 proteins and their function annotations, an RF consisting of 500 classification trees was trained to maximize the function prediction of the remaining 1,490 proteins. The trained machine was then used to predict the functions of the target protein. This procedure was repeated iteratively for all 1,491 proteins.

For each node *i*, we considered each function 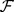 from its neighbors as a candidate function for *i*, and we wanted to design a machine to decide the adequacy of 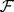. For each *i*, we calculated 11 features, 5 using only neighbor information, 5 by combining community information with the neighbor information, and one employing background information only, the fraction of proteins with function 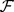. The first 5 features were 1) number of neighbors with 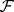, 2) difference between the numbers of neighbors with and without 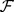, 3) fraction of neighbors with 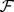, 4) rank of 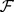 frequency among all functions from neighbors, and 5) P-value of 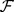 calculated from neighbors against all nodes. The next 5 features are calculated from neighbors and 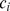, the community to which *i* belongs; 6) number of neighbors in 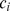, 7) ratio of 6) to all neighbors, 8) fraction of 6) with 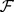, 9) P-value of 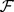 calculated from 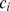 against all nodes, and 10) P-value of 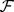 based on 6) against 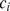.

### Evaluation of Protein Function Prediction

Prediction efficiency can be measured by precision 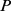 and recall 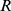, which are defined as:
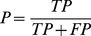
(7)

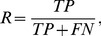
(8)where 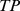 and 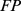 denote true and false positives and 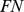 denotes false negatives.

The screening scheme inevitably leads to a smaller number of predictions than the original neighbor-based methods, which results in lower recall. However, if screening by community structure information provides significantly better prediction accuracy, and this would compensate for the loss of recall.

Another prediction quality measure used in this study is the Matthews correlation coefficient (MCC) [47], defined as:
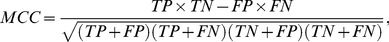
(9)where 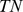 denotes true negatives. MCC measures the correlation coefficient between prediction and observation, and it returns +1 for perfect prediction, 0 for a random prediction, and −1 for perfect disagreement.

## Supporting Information

Table S1 **The complete list of genes classified by the optimal community structure of the yeast protein-complex bipartite network.**
(XLS)

Table S2 **The list of meaningful functional clusters and their P-values obtained by the optimal community structure of the protein-complex network.**
(XLS)
